# Epidemiology, Distribution and Identification of Ticks on Livestock in Pakistan

**DOI:** 10.3390/ijerph19053024

**Published:** 2022-03-04

**Authors:** Sadia Salim Khan, Haroon Ahmed, Muhammad Sohail Afzal, Mobushir Riaz Khan, Richard J. Birtles, Jonathan D. Oliver

**Affiliations:** 1 Department of Biosciences, COMSATS University Islamabad (CUI), Park Road, Chakh Shahzad, Islamabad 45550, Pakistan; sadiasalim121@gmail.com (S.S.K.); haroonahmad12@yahoo.com (H.A.); 2 Department of Life Sciences, School of Science, University of Management & Technology (UMT), Lahore 54770, Pakistan; sohail.ncvi@gmail.com; 3 School of Environmental Science, Charles Sturt University, Albury, NSW 2640, Australia; mobkhan@csu.edu.au; 4 School of Science, Engineering and Environment, University of Salford, Salford M5 4NT, UK; r.j.birtles@salford.ac.uk; 5 Environmental Health Sciences, School of Public Health, University of Minnesota, Minneapolis, MN 55455, USA

**Keywords:** livestock, ticks, tick-borne diseases

## Abstract

**Background:** Ticks are ectoparasites that transmit a variety of pathogens that cause many diseases in livestock which can result in skin damage, weight loss, anemia, reduced production of meat and milk, and mortality. **Aim:** The aim of this study was to identify tick species and the distribution on livestock hosts (sheep, goat, dairy cattle, and buffalo) of Punjab, Khyber Pakhtunkhwa Province and Islamabad from October 2019 to November 2020. **Materials and Methods:** Surveillance was performed to calculate the prevalence of ticks on livestock. Tick prevalence data (area, host, breed, gender, age, and seasonal infestation rate) was recorded and analyzed. **Results:** A total of 2080 animals were examined from selected farms, and, of these, 1129 animals were tick-infested. A total of 1010 male tick samples were identified to species using published keys. *Haemaphysalis punctata*, *Haemaphysalis sulcata*, *Hyalomma anatolicum*, *Hyalomma detritum*, *Hyalomma dromedarii*, *Hyalomma excavatum*, *Hyalomma marginatum*, *Hyalomma rufipes*, *Rhipicephalus decoloratus Rhipicephalus microplus*, and *Rhipicephalus sanguineus* were collected from goats, sheep, buffalo, and cattle. The overall rates of tick infestation on livestock were 34.83% (buffalo), 57.11% (cattle), 51.97% (sheep) and 46.94% (goats). Within each species, different breeds demonstrated different proportions of infestation. For cattle breeds, infestation proportions were as follows: Dhanni (98.73%), Jersey (70.84%) and the Australian breed of cattle (81.81%). The Neeli Ravi breed (40%) of buffalo and the Beetal breed (57.35%) of goats were the most highly infested for these species. Seasonally, the highest prevalence of infestation (76.78%) was observed in summer followed by 70.76% in spring, 45.29% in autumn, and 20% in winter. The prevalence of tick infestation in animals also varied by animal age. In goats, animals aged 4–6 years showed the highest prevalence (90%), but in cattle, the prevalence of ticks was highest (68.75%) in 6 months–1-year-old animals. 1–3 years old buffalo (41.07%) and 6 months–1 year sheep (65.78%) had the highest prevalence rate. Females had significantly higher infestation rates (61.12%, 55.56% and 49.26%, respectively) in cattle, sheep, and goats. In buffalo, males showed a higher prevalence (38.46%) rate. **Conclusions**: This study showed tick diversity, infestation rate, and numerous factors (season, age, and gender of host) influencing tick infestation rate in different breeds of cattle, sheep, goats, and buffalo in Punjab Province, Khyber Pakhtunkhwa Province, and Islamabad, Pakistan. Higher tick burdens and rates of tick-borne disease reduce production and productivity in animals. Understanding tick species’ prevalence and distribution will help to develop informed control measures.

## 1. Introduction

Pakistan is a highly agricultural country with much of its population involved in animal husbandry. Forty-three percent of workers, especially in rural areas, work in the agricultural sector [1]. Parasites, such as ticks, impact animal health and production [2,3,4]. An amount of 49.6 million cattle, 41.2 million buffalo, 78.2 million goats, and 30.9 million sheep reside in Pakistan [5]. Cattle and goats are raised all over the country in grazing areas, while most sheep are found in hilly areas and buffalo are mostly raised in Punjab and Sindh [6,7,8].

Ticks are ectoparasites that can transmit a variety of pathogens that cause many diseases in cattle. These may result in skin damage, weight loss, abortion, and mortality, leading to substantial economic losses [9,10,11]. In Pakistan, cattle, and buffalo are mostly infested with *Rhipicephalus* and *Hyalomma* ticks [7,12,13,14,15,16,17] which transmit pathogens, such as *Anaplasma marginale*, *Babesia bigemina*, *Babesia bovis* and *Theileria annulate* [18,19,20,21,22,23,24]. Ovine and caprine theileriosis are caused by *Theileria ovis* and *Theileria lestoquardi* in sheep and goats, and these pathogens are transmitted by *Haemaphysalis* and *Hyalomma* ticks [25,26,27,28,29]. Ticks may also transmit pathogens to humans, especially those working closely with animals. Crimean-Congo hemorrhagic fever virus, transmitted by ticks in the genus *Hyalomma*, is of particular concern [30,31,32].

Despite the many negative impacts on the health of livestock and the variety of tick-borne pathogens, tick infestation of livestock has been little documented in some areas of Punjab, Swat, and in Islamabad. Previous studies have examined the prevalence of tick infestation on livestock in some areas of Pakistan and they are reported in different hosts in Pakistan, e.g., sheep and goats [4,7,33] and bovines [7,14,20,21,34]. Some studies of tick prevalence in Pakistan are focused on specific regions with small sample sizes of ticks. Ticks and tick-borne diseases are neglected issues and there are few tick control products available in markets [35].

The Punjab Province of Pakistan is highly rural, and many people rely on livestock rearing for their livelihood. During the summer feasting holiday of Eid ul Azha, many herdsmen bring animals to Punjab from other provinces, and this event corresponds to an annual increase in cases of Crimean-Congo hemorrhagic fever [4,36]. It is likely that ticks carrying livestock pathogens are transferred between animal herds maintained in close quarters at this time. There is a need for appropriate strategies for the management of ticks and tick-borne diseases, and this requires current data on the prevalence and distribution of ticks on a variety of hosts. The objective of this work was to better understand the dynamics of tick-borne disease transmission among both livestock and humans in Punjab Province, Khyber Pakhtunkhwa Province (KPK), and Islamabad. To do so, we performed large-scale surveillance of tick infestation on livestock, including cattle, buffalo, sheep, and goats. This study will help to produce coherent tick control and education programs tailored to the regions. 

## 2. Materials and Methods 

### 2.1. Study Area

**Figure 1:**
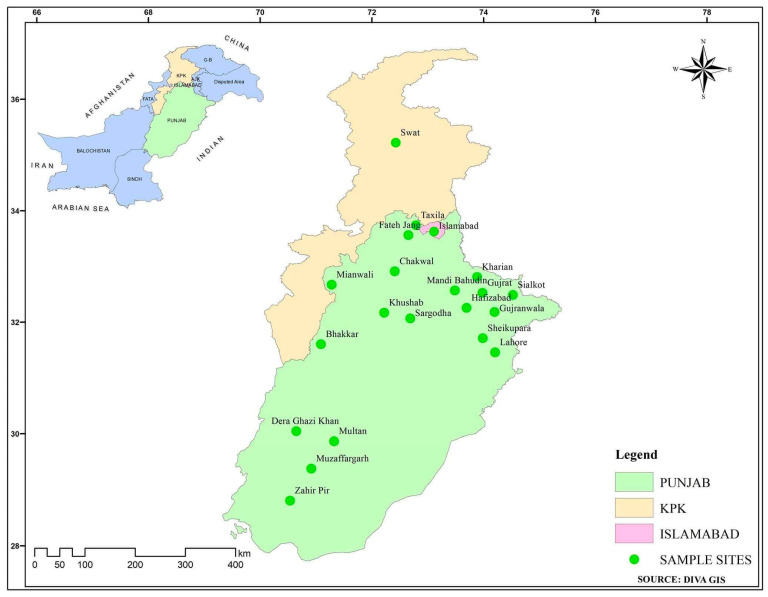
Map of Pakistan showing sampling location for this study.

This study focused on Punjab Province, Khyber Pakhtunkhwa Province and Islamabad from October 2019 to November 2020. The study sites are shown in Figure 1. Punjab Province is the most populated province of Pakistan, and this area contains 7 million sheep, 22 million buffalo, 24 million goats, and 18.8 million cattle [7,37]. The climate of Punjab is dry, as rainfall is less than 200 mm annually. The annual mean temperature of this area varies between its cold zone (7–12 °C), and its warm zone (above 25 °C) [38]. During the summer, high humidity, and temperatures provide favorable conditions for tick growth and infestation. The Swat valley is located within KPK in northern Pakistan. In its Malakand division, there are more than 80,000 sheep, 236,000 goats, 253,000 cattle, and 117,000 buffalo. The average temperature of Swat varies between 10 °C and 15 °C [39,40]. Islamabad is the capital of Pakistan. To its northeast, in Rawalpindi (Punjab), there are many livestock farms and rural areas where people rear livestock. Islamabad is the largest city in Pakistan and has a humid climate with temperatures ranging from −3.9 °C to 46. °C (January to June) [41].

### 2.2. Sample Size, Collection, and Preservation

Seventy-three livestock farms were selected for tick sampling based on the availability of permission to sample. Four villages were selected from each district shown in Figure 1. Six livestock farms were visited in each village. Farms were at least ten km away from the next farm in urban areas and more than five km away in rural areas. Livestock varying in age, breed, and sex housed in barns were selected on the supposition that if ticks are present on the farm, then at least 50% of the livestock would be infested on that farm [42]. They were checked for ticks using a standardized protocol [43]. Any ticks encountered were carefully removed with forceps to ensure they remained intact, including mouthparts [44]. Collection data regarding location, date, host species, breed, age, and sex were recorded with the help of veterinarians. Ticks were preserved in 70% ethanol. 

### 2.3. Identification of Male Ticks 

Morphological characters of ticks were observed using a Leica EZ4W stereomicroscope (Leica EZ4W) and identified using taxonomic keys [45,46,47,48].

### 2.4. Statistical Analysis 

Prevalence data (area of collection, host, breed, gender, age, and seasonal infestation rate of ticks) were analyzed by using Jomovi 1.6.23 software and statistical analysis was done in R language (version 4.0.5). The chi-square test, was calculated to assess the difference between two distributions (i.e., non-infested and tick-infested animals) and *p* < 0.05 was considered a significant level between groups [49].

## 3. Results

### 3.1. Tick Prevalence on Livestock 

A total of 2080 animals, varying in age, breed, and sex, were selected and examined for ticks. Of these, 1141 animals (31 buffalo, 791 cattle, 66 sheep, and 253 goats) were found to be tick infested. The proportion of male tick infestation was 34.83% for buffalo, 59.69% for cattle, 46.93% for goats, and 51.96% for sheep, as shown in Table 1. The unidentified female (1324) and immature ticks (531) on the four host animals (goat, sheep, buffalo, and cattle) were omitted from analysis and discussion due to the difficulty in identification. There was a statistically significant difference (*p* = 0.001, $\chi^{2}=41$) between the rates of non-infested and tick-infested animals (Table 1). 

There were high proportions of tick infestation in both indigenous and exotic breeds of cattle. Dhanni (98.73%), Jersey (70.84%), and the Australian breed of cattle (81.81%) were heavily tick-infested, while the Sahiwal breed was less infested (54.34%). In buffalo, the tick infestation rate was higher (40%) in the Neeli Ravi breed as compared to the Ravi breed. In goats, the Beetal breed was highly infested (57.35%) and Lal Puri (39.87) was least infested. There were statistically significant differences (*p* < 0.05) in tick infestation rates among some breeds of cattle ($\chi^{2}=65.8$), buffalo ($\chi^{2}=3.86$), and goats ($\chi^{2}=18.5$; Table 2).

The age of the animals affected the prevalence of tick infestation. In goats, animals aged 4–6 years showed the highest prevalence (90%) followed by 1–3 years (54.67%), 6 months–1 year (41.86%), and up to 6 months (40.83%) age groups. In cattle, the rate of tick infestation was highest (62.29%) in 4–6 years old animals, followed by 1–3 years old (60.93%), 7–14 years old (50%), 6 months–1 year (46.31%), and up to 6 months old animals (40%). In buffalo, 1–3 years (41.07%), and in sheep, 6 months–1 year (65.78%) old animals had the highest rates of tick infestation. 

In cattle, sheep, and goats, females had a higher prevalence of tick infestation (61.12%, 55.56% and 48.25%, respectively) than males (59.06%, 49.31% and 45.56%, respectively). In buffalo, males showed a higher prevalence (38.46%) of tick infestation than females (32.65%) (Table 3).

### 3.2. Geographic and Seasonal Tick Prevalence 

There were differences in tick burdens in different areas with higher infestation levels in Hafizabad (100%) and Mandi Bahudin (89.55%), and lower infestation levels in Sheikhupura (20%; Table 4).

Table 5 shows the overall seasonal tick prevalence in Punjab Province and Islamabad. The highest prevalence (76.78%) was observed in summer followed by 70.76% in spring, 45.29% in autumn, and 20% in winter. With respect to seasons, significant differences (*p* < 0.05) were observed in the proportions of non-infested and tick-infested animals.

### 3.3. Tick Species Identification

Tick samples (males and females of different life stages) were collected from 1141 animals (sheep, goats, cattle, and buffalo) from different areas of Punjab Province, KPK, and Islamabad. An amount of 1012 male ticks were found and identified to species. Because the ticks were collected from livestock, most immatures and females were engorged, obscuring diagnostic characters. This was particularly of concern for *Hyalomma* ticks for which diagnostic characters often include the shape of the genital aperture [47]. Eleven species of ticks (*H. punctata*, *H. sulcata*, *H. anatolicum*, *H. detritum*, *H. dromedarii*, *H. excavatum*, *H. marginatum*, *H. rufipes*, *R. decoloratus*, *R. microplus*, and *R. sanguineus*) were identified. Only goats and cattle were infested with *H. dromedarii*, and *H. sulcata* was only identified on buffalo and sheep (Table 6). Table 7 shows the tick species (male) found on various animals.

## 4. Discussion

Tick identification was performed morphologically using dichotomous keys. One potential shortcoming of this project was the identification of only male ticks. Eleven tick species in three genera were identified. Male ticks of these species all feed on livestock and are implicated in the transmission of tick-borne diseases. In areas where resources for molecular identification of tick species are not available, researchers must rely on traditional morphological identification methods. It will be valuable to develop regionally specific identification keys that reflect all species, ages, and sexes of ticks likely to be encountered. Some areas of Punjab, Swat, and Islamabad had the highest diversity of tick species observed amongst livestock at a given site in Pakistan. The diversity of tick species observed may be due to the collection of ticks from a variety of livestock hosts. Identified species included several important disease vectors, e.g., *Rhipicephalus*, *Hyalomma* [7,12,13,50,51] and *Haemaphysalis* [27,28,29].

The highest proportion of livestock infested with ticks was observed in cattle. This may be due to their thin skin and the favorable habitat and climatic conditions for ticks in Pakistan [20]. Our results regarding lower tick infestation rates in buffalo as compared to cattle are similar to the previous findings that reported a lower prevalence of tick infestation in buffalo than in cattle [4,21,34]. 

The tick infestation rate was higher in sheep than in goats. This pattern has been observed in some studies of tick infestation of livestock in Pakistan [52,53]. Another study observed the opposite: that infestation was lower in sheep (11.1%) as compared to goats (60.0%) [7]. The lower rate of tick infestation in goats was maybe due to pasturing in steep and rocky habitats that limit contact with other species of livestock [54].

Exotic livestock breeds and their crosses have longer and denser hair, which makes them an easy victim of tick infestation due to extensive sheltered attachment space. The lower infestation rate in indigenous breeds (Sahiwal cattle) was also may be due to the development of resistance due to constant exposure to parasites [9,55]. Higher rates of tick infestation in exotic breeds of cattle have been noted in previous studies in Pakistan [7,12,13,14,55,56,57] as well as in Egypt [58]. Previous studies [4,24] also reported higher tick infestation rates in local breeds of cattle, which is also observed in our study, as the Dhanni breed of cattle were highly infested with ticks. Our results regarding the higher rate of tick infestation in crossbred cattle disagree with several other studies that observed a lower rate of infestation in cross breeds [34,56,59] 

The higher rates of tick infestation observed in the summer are likely due to the combination of increased moisture and higher temperature [48,60]. Previous studies have shown peak infestation rates occurring in the summer [57,61,62] or during other seasons [63,64] depending on local climatic and environmental conditions, such as humidity, temperature, and host animal availability [65,66,67]. As large-bodied ticks, *Hyalomma* is generally more resistant to desiccation and some species, such as *H. dromedarii*, are well adapted to living in dry, even desert habitats [68].

Animal age had an important effect on the prevalence of ticks [69]. Young and adult cattle were heavily infested with ticks as compared to calves as has been previously reported [7,24,57]. Grooming of calves and the smaller surface area of animals may be factors in the lower tick burdens [70]. In sheep and goats, age had no significant effect on tick infestation, as others have described [53].

Female cattle, sheep, and goats were slightly more likely to have ticks than males. This result is contrary to some previous studies [56,71]. In buffalo, males were more likely to be infested than females, as has been previously described [21]. 

## 5. Conclusions

This study described the prevalence of tick infestation in cattle, sheep, goats, and buffalo in Punjab Province, Khyber Pakhtunkhwa Province, and Islamabad. Eleven tick species that can transmit a variety of pathogens to livestock and humans were identified. This information will help develop locally appropriate tick control and education programs in the region.

## Figures and Tables

**Table 1:** Host wise prevalence rate of male ticks.

Hosts	Non-Infested	Tick-Infested	Total	Prevalence (%)
Buffalo	58	31	89	34.83
Cattle	534	791	1325	59.69
Goat	286	253	539	46.93
Sheep	61	66	127	51.96
Chi-square test (*p*-value)	41 (0.001 *)			

* Significant difference (*p* < 0.05).

**Table 2:** Breed wise prevalence rate of male ticks.

Hosts	Breeds	Non-Infested	Tick-Infested	Total	Prevalence (%)
Cattle	Australian	12	54	66	81.81
	Cross breed	39	97	136	71.32
	Dhanni	1	78	79	98.73
	Freisian	181	217	398	54.52
	Sahiwal	284	338	622	54.34
	Jersey	7	17	24	70.84
Chi-square test (*p*-value)	65.8 (0.001 *)				
Buffalo	Neeli Ravi	42	28	70	40
	Ravi	16	3	19	15.78
Chi-square test (*p*-value)	3.86 (0.049 *)				
Sheep	Pure Kajla	61	66	127	51.96
Goats	Rajanpuri	34	26	60	43.34
	Beetal	58	78	136	57.35
	Desi Teddy	109	76	185	41.08
	Lal puri	95	63	158	39.87
Chi-square test (*p*-value)	18.5 (0.001 *)				

* Significant difference (*p* < 0.05).

**Table 3:** Prevalence of male tick infestation in relation to age and gender.

Host	S	Up to 6 Months				6 Month–1 Year				1–3 Years				4–6 Years				7–14 Years				N%
		+	−	N	%	+	−	N	%	+	−	N	%	+	-ve	N	%	+	−	N	%	
Buffalo	M	0	0	0	0	6	16	22	27.27	9	8	17	52.94	0	0	0	0	0	0	0	0	38.46
	F	0	0	0	0	2	8	10	20	14	25	39	35.89	0	0	0	0	0	0	0	0	32.65
%		0				25				41.07				0				0				
Goats	M	35	39	74	47.29	77	99	176	41.2	36	45	81	44.44	6	1	7	85.71	0	0	0	0	45.56
	F	14	32	46	30.43	31	51	82	37.8	40	18	58	68.96	12	1	13	92.3	0	2	2	0	48.25
%		40.83				41.86				54.67				90				0				
Cattle	M	3	7	10	30	35	40	75	46.66	485	321	806	60.17	17	7	24	70.83	1	0	1	100	59.06
	F	3	2	5	60	9	11	20	45	217	129	346	62.71	21	16	37	56.75	0	1	1	0	61.12
%		40				46.31				60.93				62.29				50				
Sheep	M	25	28	53	47.16	9	7	16	56.25	2	2	4	50	0	0	0	0	0	0	0	0	49.31
	F	13	17	30	43.33	16	6	22	72.72	1	1	2	50	0	0	0	0	0	0	0	0	55.56
%		45.78				65.78				50				0				0				

S = Sex, M = Male, F = Female, + = Tick infested, − = Non-Infested, N = Total, % = Prevalence, N% = Total Prevalence.

**Table 4:** Area wise prevalence rate of male ticks.

Area	Latitude N	Longitude E	Non-Infested	Tick Infested	Total	Prevalence (%)
Bhakkar	31.6082	71.0854	8	14	22	63.63
Chakwal	32.9172	72.4081	14	106	120	88.34
Dera Ghazi Khan	30.0489	70.6455	26	16	42	38.09
Fateh Jang	33.5673	72.6506	32	35	67	52.23
Gujranwala	32.1877	74.1945	4	15	19	78.94
Gujrat	32.5295	73.9771	36	26	62	41.93
Hafizabad	32.2623	73.6945	0	30	30	100
Islamabad	33.6296	73.1123	73	124	197	62.94
Kharian	32.8143	73.8831	94	116	210	55.23
Khushab	32.1748	72.219	54	61	115	53.04
Kunjah	32.5295	73.9771	10	21	31	67.74
Lahore	31.4635	74.2047	155	168	323	52.01
Mandi Bahudin	32.5742	73.4828	7	60	67	89.55
Mianwali	32.6749	71.2785	61	41	102	40.19
Multan	29.8717	71.3231	47	41	88	46.59
Makwal	30.5851	70.7258	82	81	163	49.69
Muzaffargarh	29.3817	70.9131	15	5	20	25
Sargodha	32.074	72.6861	2	13	15	86.67
Sheikupara	31.7167	73.985	24	6	30	20
Sialkot	32.4945	74.5229	12	23	35	65.71
Swat	35.2227	72.4258	56	38	94	40.42
Talagang	32.9172	72.4081	15	7	22	31.81
Taunsa	30.7046	70.6574	11	12	23	52.17
Taxila	33.7408	72.7858	58	50	108	46.29
Zahir Pir	28.8107	70.5324	43	32	75	42.67

**Table 5:** Seasonal prevalence rate of male ticks.

Season	Non-Infested	Tick Infested	Total	Prevalence (%)	Chi-Square (χ^2^)	*p*-Value
Autumn	756	626	1382	45.29	189	0.001 *
Summer	140	463	603	76.78		
Winter	24	6	30	20		
Spring	19	46	65	70.76		

* Significant (*p* < 0.05).

**Table 6:** Number of male ticks by species found in Punjab, Islamabad, and KPK, Pakistan.

Province	Areas	Tick Species										
		*Haemaphysalis punctata*	*Haemaphysalis sulcata*	*Hyalomma dromedarii*	*Hyalomma anatolicum*	*Hyalomma detritum*	*Hyalomma excavatum*	*Hyalomma marginatum*	*Hyalomma rufipes*	*Rhipicephalus microplus*	*Rhipicephalus decoloratus*	*Rhipicephalus sanguineus*
**Punjab**	Mandi Bahudin	1	0	0	41	0	0	3	0	3	2	6
	Kharian	1	0	1	12	3	0	4	0	4	3	2
	Dera Ghazi Khan	3	1	1	8	24	10	12	0	1	2	45
	Muzaffargarh	0	0	0	4	0	0	1	0	1	1	10
	Mianwali	0	0	1	6	4	4	03	0	0	0	3
	Zahir Pir	0	0	2		5	3	1	0	0	0	12
	Fateh Jang	0	0	0	10	0	1	2	0	4	1	4
	Sheikupara	0	0	0	1	0	0	1	0	8	0	0
	Lahore	0	0	0	45	35	6	10	0	0	3	0
	Chakwal	0	0	0	71	25	1	41	1	10	2	0
	Sargodha	0	0	0	10	0	0	0	0	1	3	0
	Hafizabad	0	0	0	16	0	0	0	0	0	1	0
	Gujrat	0	0	0	19	2	7	2	0	3	0	0
	Bhakkar	0	0	0	1	0	0	0	0	2	1	0
	Multan	0	0	0	1	0	0	0	0	0	0	0
	Taxila	0	0	0	7	0	3	0	0	0	0	0
	Sialkot	0	0	0	0	1	0	1	0	2	2	0
**Islamabad**	Islamabad	3	1	0	30	4	0	20	0	6	0	303
**KPK**	Swat	4	0	0	0	2	0	4	0	0	1	8
	Total	**12**	**2**	**5**	**287**	**105**	**35**	**105**	**1**	**45**	**22**	**393**

**Table 7:** Number of tick species (males) found on various animals.

Tick Species	Goat	Sheep	Buffalo	Cattle
*Haemaphysalis punctata*	11	1	0	0
*Rhipicephalus sanguineus*	350	34	6	3
*Haemaphysalis sulcata*	0	0	2	0
*Hyalomma anatolicum*	27	9	8	243
*Hyalomma detritum*	44	18	1	42
*Hyalomma excavatum*	8	3	1	23
*Hyalomma marginatum*	21	13	0	71
*Rhipicephalus microplus*	2	1	0	42
*Rhipicephalus decoloratus*	2	0	1	19
*Hyalomma rufipes*	0	0	0	1
*Hyalomma dromedarii*	4	0	0	1

## Data Availability

Specimens of female and immature ticks have been retained at the University of Minnesota Division of Environmental Health Sciences and are available for study.
